# Supplementary material for: Serum anti-nucleocapsid antibody correlates of protection from SARS-CoV-2 re-infection regardless of symptoms or immune history
Source: Commun Med (Lond). 2025 May 15;5:172. doi: 10.1038/s43856-025-00894-8 (PMC12081900; doi:10.1038/s43856-025-00894-8)
Supplement: Supplementary file 2 — Supplementary Information [file 43856_2025_894_MOESM2_ESM.pdf]

Serum anti-nucleocapsid antibody correlates of protection from SARS-CoV-2 re-infection regardless of symptoms or immune history

Sho Miyamoto, Koki Numakura, Ryo Kinoshita, Takeshi Arashiro, Hiromizu Takahashi, Hiromi Hibino, Minako Hayakawa, Takayuki Kanno, Akiko Sataka, Rena Sakamoto, Akira Ainai, Satoru Arai, Motoi Suzuki, Daisuke Yoneoka, Takaji Wakita, Tadaki Suzuki

Supplementary Information

Supplementary Table 1. Characteristics of individuals with and without infection during the study period.

	Newly infected (n = 389) Median (IQR); n (%)	95% CI	Non-infected (n = 4,107) Median (IQR); n (%)	95% CI
Age in years				
20-29	20 (5.1%)	3.2 – 7.8%	133 (3.2%)	2.7 – 3.8%
30-39	52 (13.4%)	10.1 – 17.2%	381 (9.3%)	8.4 – 10.2%
40-49	108 (27.8%)	23.4 – 32.5%	830 (20.2%)	19.0 – 21.5%
50-59	104 (26.7%)	22.4 – 31.4%	1,079 (26.3%)	25.0 – 27.7%
60-69	65 (16.7%)	13.1 – 20.8%	842 (20.5%)	19.3 – 21.8%
70-79	31 (8.0%)	5.5 – 11.1%	634 (15.5%)	14.4 – 16.6%
80-	9 (2.3%)	1.1 – 4.4%	204 (5.0%)	4.3 – 5.7%
No response	0		4	
Sex				
Female	259 (66.6%)	61.7 – 71.3%	2,521 (61.5%)	60.0 – 63.0%
Male	130 (33.4%)	28.7 – 38.3%	1,579 (38.5%)	37.0 – 40.0%
Other/no response	0		7	
Prefecture				
Miyagi	67 (17.2%)	13.6 – 21.4%	896 (21.8%)	20.6 – 23.1%
Tokyo	82 (21.1%)	17.1 – 25.5%	1,057 (25.7%)	24.4 – 27.1%
Aichi	85 (21.9%)	17.8 – 26.3%	729 (17.8%)	16.6 – 19.0%
Osaka	88 (22.6%)	18.6 – 27.1%	723 (17.6%)	16.5 – 18.8%
Fukuoka	67 (17.2%)	13.6 – 21.4%	702 (17.1%)	16.0 – 18.3%
Comorbidity				
No comorbidity	222 (58.3%)	53.1 – 63.3%	2,059 (51.4%)	49.8 – 52.9%
Asthma	9 (2.4%)	1.1 – 4.4%	65 (1.6%)	1.3 – 2.1%
Cancer	6 (1.6%)	0.6 – 3.4%	69 (1.7%)	1.3 – 2.2%
Chronic obstructive pulmonary disease	0 (0.0%)	0.0 – 1.0%	7 (0.2%)	0.1 – 0.4%
Diabetes mellitus	10 (2.6%)	1.3 – 4.8%	120 (3.0%)	2.5 – 3.6%
Heart disease	6 (1.6%)	0.6 – 3.4%	63 (1.6%)	1.2 – 2.0%
Hypertension	41 (10.8%)	7.8 – 14.3%	556 (13.9%)	12.8 – 15.0%
Immunodeficiency/immunosuppressant use	0 (0.0%)	0.0 – 1.0%	24 (0.6%)	0.4 – 0.9%
Kidney disease	2 (0.5%)	0.1 – 1.9%	13 (0.3%)	0.2 – 0.6%
Liver disease	3 (0.8%)	0.2 – 2.3%	15 (0.4%)	0.2 – 0.6%
Obesity	2 (0.5%)	0.1 – 1.9%	30 (0.7%)	0.5 – 1.1%
Combined	25 (6.6%)	4.3 – 9.5%	394 (9.8%)	8.9 – 10.8%
Others	55 (14.4%)	11.1 – 18.4%	593 (14.8%)	13.7 – 15.9%
No response	8		99	
Vaccination status				
None	19 (4.9%)	3.0 – 7.5%	143 (3.5%)	2.9 – 4.1%
Once	1 (0.3%)	0.0 – 1.4%	5 (0.1%)	0.0 – 0.3%
Twice	32 (8.2%)	5.7 – 11.4%	260 (6.3%)	5.6 – 7.1%
Three times	110 (28.3%)	23.9 – 33.0%	844 (20.6%)	19.3 – 21.8%
Four times	132 (33.9%)	29.2 – 38.9%	1,378 (33.6%)	32.1 – 35.0%
Five times	95 (24.4%)	20.2 – 29.0%	1,477 (36.0%)	34.5 – 37.5%
Vaccine type				
Bivalent (BA.1 or BA.5)	200 (51.4%)	46.3 – 56.5%	2,630 (64.0%)	62.5 – 65.5%
Ancestral monovalent	170 (43.7%)	38.7 – 48.8%	1,334 (32.5%)	31.0 – 33.9%

Unvaccinated	19 (4.9%)	3.0 – 7.5%	143 (3.5%)	2.9 – 4.1%
Prior infection history/period				
No prior-infection	350 (90.0%)	86.5 – 92.8%	3,081 (75.0%)	73.7 – 76.3%
Omicron (after Jan. 2022)	10 (2.6%)	1.2 – 4.7%	616 (15.0%)	13.9 – 16.1%
Pre-Omicron (before Dec. 2021)	1 (0.3%)	0.0 – 1.4%	30 (0.7%)	0.5 – 1.0%
Prior-infection with unknown date	28 (7.2%)	4.8 – 10.2%	380 (9.3%)	8.4 – 10.2%
Hybrid immunity				
Hybrid	36 (9.3%)	6.57 – 12.6%	954 (23.2%)	21.9 – 24.6%
Non-hybrid	353 (90.7%)	87.4 – 93.4%	3,153 (76.8%)	75.4 – 78.1%
Days from the first to the second test	63 (56 – 70)		62 (56 – 69)	
Days from last vaccination to first test	23 (12 – 48)		35 (17 – 63)	
Missing/no response	381		3,796	
Days from the last infection to the first test	192 (162 – 223)		121 (82 – 147)	
Missing/no response	387		3,827	
Days from last exposure (vaccination or infection) to first test	77 (30 – 252)		42 (24 – 124)	
Missing/no response	86		818	

Supplementary Table 2. Characteristics of newly infected cases during the study period.

	Non-hybrid (n = 353) n (%)	95% CI	Hybrid (n = 36) n (%)	95% CI
Age in years				
20-29	17 (4.8%)	2.8 – 7.6%	3 (8.3%)	1.8 – 22.5%
30-39	47 (13.3%)	10.0 – 17.3%	5 (13.9%)	4.7 – 29.5%
40-49	96 (27.2%)	22.6 – 32.2%	12 (33.3%)	18.6 – 51.0%
50-59	96 (27.2%)	22.6 – 32.2%	8 (22.2%)	10.1 – 39.2%
60-69	59 (16.7%)	13.0 – 21.0%	6 (16.7%)	6.4 – 32.8%
70-79	30 (8.5%)	5.8 – 11.9%	1 (2.8%)	0.1 – 14.5%
80-	8 (2.3%)	1.0 – 4.4%	1 (2.8%)	0.1 – 14.5%
Sex				
Female	236 (66.9%)	61.7 – 71.7%	23 (63.9%)	46.2 – 79.2%
Male	117 (33.1%)	28.3 – 38.3%	13 (36.1%)	20.8 – 53.8%
Prefecture				
Miyagi	65 (18.4%)	14.5 – 22.9%	2 (5.6%)	0.7 – 18.7%
Tokyo	75 (21.2%)	17.1 – 25.9%	7 (19.4%)	8.2 – 36.0%
Aichi	76 (21.5%)	17.4 – 26.2%	9 (25%)	12.1 – 42.2%
Osaka	77 (21.8%)	17.6 – 26.5%	11 (30.6%)	16.3 – 48.1%
Fukuoka	60 (17%)	13.2 – 21.3%	7 (19.4%)	8.2 – 36.0%
Vaccination status				
None	19 (5.4%)	3.27 – 8.28%	0 (0.0%)	0.0 – 9.7%
Once	1 (0.3%)	0.0 – 1.6%	0 (0.0%)	0.0 – 9.7%
Twice	30 (8.5%)	5.8 – 11.9%	2 (5.6%)	0.7 – 18.7%
Three times	97 (27.5%)	22.9 – 32.5%	13 (36.1%)	20.8 – 53.8%
Four times	121 (34.3%)	29.3 – 39.5%	11 (30.6%)	16.3 – 48.1%
Five times	85 (24.1%)	19.7 – 28.9%	10 (27.8%)	14.2 – 45.2%
Comorbidity				
No comorbidity	195 (56.5%)	51.1 – 61.8%	27 (75%)	57.8 – 87.9%
Asthma	9 (2.6%)	1.2 – 4.9%	0 (0.0%)	0.0 – 9.7%
Cancer	6 (1.7%)	0.6 – 3.8%	0 (0.0%)	0.0 – 9.7%
Chronic obstructive pulmonary disease	0 (0.0%)	0.0 – 1.0%	0 (0.0%)	0.0 – 9.7%
Diabetes mellitus	9 (2.6%)	1.2 – 4.9%	1 (2.8%)	0.1 – 14.5%
Heart disease	6 (1.7%)	0.6 – 3.8%	0 (0.0%)	0.0 – 9.7%
Hypertension	39 (11.3%)	8.2 – 15.1%	2 (5.6%)	0.7 – 18.7%
Immunodeficiency/immunosuppressant use	0 (0.0%)	0.0 – 1.0%	0 (0.0%)	0.0 – 9.7%
Kidney disease	2 (0.6%)	0.1 – 2.1%	0 (0.0%)	0.0 – 9.7%
Liver disease	2 (0.6%)	0.1 – 2.1%	1 (2.8%)	0.1 – 14.5%
Obesity	1 (0.3%)	0.0 – 1.6%	1 (2.8%)	0.1 – 14.5%
Combined	25 (7.2%)	4.7 – 10.5%	0 (0.0%)	0.0 – 9.7%
Others	51 (14.8%)	11.2 – 19.0%	4 (11.1%)	3.1 – 26.1%
No response	8		0	
Vaccine type				
Bivalent (BA.1 or BA.5)	178 (50.4%)	45.1 – 55.8%	22 (61.1%)	43.5 – 76.9%
Ancestral monovalent	156 (44.2%)	38.9 – 49.5%	14 (38.9%)	23.1 – 56.5%
Unvaccinated	19 (5.4%)	3.3 – 8.3%	0 (0.0%)	0.0 – 9.7%
Prior infection history				

No prior-infection	350 (99.2%)	97.5 – 99.8%	0 (0.0%)	0.0 – 9.7%
Prior-infection	3 (0.8%)	0.2 – 2.5%	36 (100%)	90.3 – 100.0%
Newly diagnosis				
Newly diagnosed	180 (51%)	45.6 – 56.3%	2 (5.6%)	0.7 – 18.7%
Non newly diagnosed	173 (49%)	43.7 – 54.4%	34 (94.4%)	81.3 – 99.3%
Newly anti-N positive (≥1 COI)				
Already anti-N positive	2 (0.6%)	0.1 – 2.0%	35 (97.2%)	85.5 – 99.9%
Newly anti-N positive	341 (96.6%)	94.1 – 98.2%	1 (2.8%)	0.1 – 14.5%
Anti-N negative	10 (2.8%)	1.4 – 5.2%	0 (0.0%)	0.0 – 9.7%
Anti-N fold increase				
≥4 fold increase	350 (99.2%)	97.5 – 99.8%	35 (97.2%)	85.5 – 99.9%
<4 fold increase	3 (0.8%)	0.2 – 2.5%	1 (2.8%)	0.1 – 14.5%
Symptom status				
Symptomatic	167 (47.3%)	42.0 – 52.7%	2 (5.6%)	0.7 – 18.7%
Asymptomatic SARS-CoV-2 test-confirmed	12 (3.4%)	1.877 – 5.9%	0 (0.0%)	0.0 – 9.7%
Asymptomatic serological defined	174 (49.3%)	44.0 – 54.6%	34 (94.4%)	81.3 – 99.3%

Supplementary Table 3. Characteristics of individuals with symptomatic and/or asymptomatic infection during the study period.

	**Overall** (N = 4,496) n (%)	**Newly infected (symptomatic& asymptomatic)** (N = 389) n (%)	**Newly symptomatic Infected** (N = 169) n (%)	**Overall excluding symptomatic infected** (N = 4,327) n (%)	**Newly asymptomatic Infected** (N = 220) n (%)
**Age in years**					
20-29	153 (3.4%)	20 (5.1%)	9 (5.3%)	144 (3.3%)	11 (5.0%)
30-39	433 (9.6%)	52 (13.4%)	24 (14.2%)	409 (9.5%)	28 (12.7%)
40-49	938 (20.9%)	108 (27.8%)	48 (28.4%)	890 (20.6%)	60 (27.3%)
50-59	1,183 (26.3%)	104 (26.7%)	53 (31.4%)	1,130 (26.1%)	51 (23.2%)
60-69	907 (20.2%)	65 (16.7%)	24 (14.2%)	883 (20.4%)	41 (18.6%)
70-79	665 (14.8%)	31 (8.0%)	8 (4.7%)	657 (15.2%)	23 (10.5%)
80-	213 (4.7%)	9 (2.3%)	3 (1.8%)	210 (4.9%)	6 (2.7%)
No response	4	0	0	4	0
**Sex**					
Female	2,780 (61.9%)	259 (66.6%)	118 (69.8%)	2,662 (61.6%)	141 (64.1%)
Male	1,709 (38.1%)	130 (33.4%)	51 (30.2%)	1,658 (38.4%)	79 (35.9%)
Other/no response	7	0	0	7	0
**Vaccination status**					
None	162 (3.6%)	19 (4.9%)	11 (6.5%)	151 (3.5%)	8 (3.6%)
Once	6 (0.1%)	1 (0.3%)	0 (0.0%)	6 (0.1%)	1 (0.5%)
Twice	292 (6.5%)	32 (8.2%)	18 (10.7%)	274 (6.3%)	14 (6.4%)
Three times	954 (21.2%)	110 (28.3%)	52 (30.8%)	902 (20.8%)	58 (26.4%)
Four times	1,510 (33.6%)	132 (33.9%)	56 (33.1%)	1,454 (33.6%)	76 (34.5%)
Five times	1,572 (35.0%)	95 (24.4%)	32 (18.9%)	1,540 (35.6%)	63 (28.6%)
**Vaccine type**					
Bivalent (BA.1 or BA.5)	2,830 (62.9%)	200 (51.4%)	78 (46.2%)	2,752 (63.6%)	122 (55.5%)
Ancestral monovalent	1,504 (33.5%)	170 (43.7%)	80 (47.3%)	1,424 (32.9%)	90 (40.9%)
Unvaccinated	162 (3.6%)	19 (4.9%)	11 (6.5%)	151 (3.5%)	8 (3.6%)
**Prior infection history**					
No prior-infection	3,431 (76.3%)	350 (90.0%)	167 (98.8%)	3,264 (75.4%)	183 (83.2%)
Prior-infection	1,065 (23.7%)	39 (10.0%)	2 (1.2%)	1,063 (24.6%)	37 (16.8%)
**Hybrid immunity**					
Hybrid	990 (22.0%)	36 (9.3%)	2 (1.2%)	988 (22.8%)	34 (15.5%)
Non hybrid	3,506 (78.0%)	353 (90.7%)	167 (98.8%)	3,339 (77.2%)	186 (84.5%)

Supplementary Table 4. Characteristics of individuals in the subsampling cohort for neutralizing antibody response analysis.

		Hybrid		Non-hybrid	
	**Subsampling** (N = 80) n (%)	**Newly infected** (N = 20) n (%)	**Non-infected** (N = 20) n (%)	**Newly infected** (N = 20) n (%)	**Non-infected** (N = 20) n (%)
**Age in years**					
20-29	6 (7.5%)	1 (5.0%)	2 (10.0%)	1 (5.0%)	2 (10.0%)
30-39	14 (17.5%)	2 (10.0%)	4 (20.0%)	4 (20.0%)	4 (20.0%)
40-49	20 (25.0%)	6 (30.0%)	3 (15.0%)	6 (30.0%)	5 (25.0%)
50-59	23 (28.8%)	4 (20.0%)	6 (30.0%)	5 (25.0%)	8 (40.0%)
60-69	14 (17.5%)	5 (25.0%)	4 (20.0%)	4 (20.0%)	1 (5.0%)
70-79	2 (2.5%)	1 (5.0%)	1 (5.0%)	0 (0.0%)	0 (0.0%)
80-	1 (1.3%)	1 (5.0%)	0 (0.0%)	0 (0.0%)	0 (0.0%)
**Sex**					
Female	52 (65.0%)	10 (50.0%)	11 (55.0%)	15 (75.0%)	16 (80.0%)
Male	28 (35.0%)	10 (50.0%)	9 (45.0%)	5 (25.0%)	4 (20.0%)
**Vaccination status**					
None	2 (2.5%)	0 (0.0%)	0 (0.0%)	1 (5.0%)	1 (5.0%)
Twice	11 (13.8%)	2 (10.0%)	2 (10.0%)	4 (20.0%)	3 (15.0%)
Three times	18 (22.5%)	2 (10.0%)	2 (10.0%)	6 (30.0%)	8 (40.0%)
Four times	29 (36.3%)	7 (35.0%)	9 (45.0%)	7 (35.0%)	6 (30.0%)
Five times	20 (25.0%)	9 (45.0%)	7 (35.0%)	2 (10.0%)	2 (10.0%)
**Vaccine type**					
Bivalent (BA.1 or BA.5)	45 (56.3%)	15 (75.0%)	16 (80.0%)	7 (35.0%)	7 (35.0%)
Ancestral monovalent	33 (41.3%)	5 (25.0%)	4 (20.0%)	12 (60.0%)	12 (60.0%)
Unvaccinated	2 (2.5%)	0 (0.0%)	0 (0.0%)	1 (5.0%)	1 (5.0%)
**Prior infection history**					
No prior-infection	39 (48.8%)	0 (0.0%)	0 (0.0%)	20 (100.0%)	19 (95.0%)
Prior-infection	41 (51.3%)	20 (100.0%)	20 (100.0%)	0 (0.0%)	1 (5.0%)
**Hybrid immunity**					
Hybrid	40 (50.0%)	20 (100.0%)	20 (100.0%)	0 (0.0%)	0 (0.0%)
Non hybrid	40 (50.0%)	0 (0.0%)	0 (0.0%)	20 (100.0%)	20 (100.0%)

Supplementary Table 5. Characteristics of individuals in the subsampling cohort for infection risk estimation using BA.5 neutralizing antibody titer.

	**Overall cohort** (N = 4,496) n (%)	**Subsampling** (N = 340) n (%)
**Age in years**		
20-29	153 (3.4%)	15 (4.4%)
30-39	433 (9.6%)	40 (11.8%)
40-49	938 (20.9%)	69 (20.3%)
50-59	1,183 (26.3%)	100 (29.4%)
60-69	907 (20.2%)	72 (21.2%)
70-79	665 (14.8%)	32 (9.4%)
80-	213 (4.7%)	12 (3.5%)
No response	4	0
**Sex**		
Female	2,780 (61.9%)	208 (61.2%)
Male	1,709 (38.1%)	132 (38.8%)
Other/no response	7	0
**Vaccination status**		
None	162 (3.6%)	14 (4.1%)
Once	6 (0.1%)	0 (0.0%)
Twice	292 (6.5%)	25 (7.4%)
Three times	954 (21.2%)	75 (22.1%)
Four times	1,510 (33.6%)	133 (39.1%)
Five times	1,572 (35.0%)	93 (27.4%)
**Vaccine type**		
Bivalent (BA.1 or BA.5)	2,830 (62.9%)	210 (61.8%)
Ancestral monovalent	1,504 (33.5%)	116 (34.1%)
Unvaccinated	162 (3.6%)	14 (4.1%)
**Prior infection history**		
No prior-infection	3,431 (76.3%)	258 (75.9%)
Prior-infection	1,065 (23.7%)	82 (24.1%)
**Hybrid immunity**		
Hybrid	990 (22.0%)	73 (21.5%)
Non hybrid	3,506 (78.0%)	267 (78.5%)

Supplementary Table 6. Summary of the model estimates from total infection risk estimation using anti-S and anti-N antibody titers.

Parameter	Estimate	Standard Error	95% CI	Rhat	Effective sample size
**Symptomatic and asymptomatic**					
Intercept	-2.95	0.02	-2.99, -2.91	1.00	3142
Log_{10} anti-S	0.81	0.08	0.66, 0.97	1.00	3367
Log_{10} anti-N	5.73	0.22	5.31, 6.16	1.00	3151
SD_{anti-S}	1.81	1.64	0.34, 6.24	1.00	2714
SD_{anti-N}	4.08	2.57	1.57, 10.59	1.00	3070
**Symptomatic**					
Intercept	-4.35	0.06	-4.48, -4.24	1.00	3020
Log_{10} anti-S	1.44	0.12	1.21, 1.68	1.00	3043
Log_{10} anti-N	5.72	0.85	4.35, 7.54	1.00	2963
SD_{anti-S}	2.80	1.96	0.88, 8.09	1.00	2787
SD_{anti-N}	1.76	1.98	0.07, 6.36	1.00	2580
**Asymptomatic**					
Intercept	-3.40	0.02	-3.44, -3.35	1.00	3079
Log_{10} anti-S	0.59	0.11	0.37, 0.80	1.00	3307
Log_{10} anti-N	5.43	0.23	4.99, 5.87	1.00	3051
SD_{anti-S}	1.01	1.41	0.02, 5.02	1.00	2270
SD_{anti-N}	4.19	2.42	1.66, 9.96	1.00	3095

SD, standard derivation

Supplementary Table 7. Summary of the model estimates from total infection risk estimation using anti-S and anti-N antibody titers in the subsampling population.

Parameter	Estimate	Standard Error	95% CI	Rhat	Effective sample size
**Symptomatic and asymptomatic**					
Intercept	-3.04	0.08	-3.20, -2.90	1.00	3099
Log_{10} anti-S	1.08	0.28	0.50, 1.59	1.00	2979
Log_{10} anti-N	5.81	0.82	4.32, 7.51	1.00	3173
SD_{anti-S}	2.27	1.71	0.53, 7.00	1.00	2979
SD_{anti-N}	3.63	2.25	1.27, 9.73	1.00	2725
**Symptomatic**					
Intercept	-5.47	0.59	-6.94, -4.64	1.00	2597
Log_{10} anti-S	1.96	0.42	1.11, 2.72	1.00	2702
Log_{10} anti-N	11.62	5.58	4.71, 26.30	1.00	2253
SD_{anti-S}	4.01	2.26	1.55, 10.17	1.00	2999
SD_{anti-N}	2.40	2.32	0.08, 8.36	1.00	2608
**Asymptomatic**					
Intercept	-3.46	0.09	-3.64, -3.29	1.00	3181
Log_{10} anti-S	0.66	0.40	-0.18, 1.37	1.00	3067
Log_{10} anti-N	5.70	0.88	4.09, 7.52	1.00	3078
SD_{anti-S}	1.28	1.49	0.03, 5.51	1.00	2650
SD_{anti-N}	3.83	2.56	1.38, 10.51	1.00	2878

SD, standard derivation

Supplementary Table 8. Summary of the model estimates from total infection risk estimation using BA.5 neutralizing and anti-N antibody titers in the subsampling population.

Parameter	Estimate	Standard Error	95% CI	Rhat	Effective sample size
**Symptomatic and asymptomatic**					
Intercept	-3.04	0.08	-3.20, -2.89	1.00	3145
Log_{10} BA.5 NT	0.83	0.22	0.40, 1.28	1.00	3187
Log_{10} anti-N	5.68	0.85	4.12, 7.35	1.00	3167
$SD_{BA.5\ NT}$	1.37	1.54	0.09, 5.02	1.00	2599
$SD_{anti\text{-}N}$	3.60	2.46	1.21, 9.93	1.00	2866
**Symptomatic**					
Intercept	-5.50	0.65	-7.04, -4.62	1.00	2645
Log_{10} BA.5 NT	3.36	0.80	1.95, 5.23	1.00	3155
Log_{10} anti-N	10.87	6.14	3.81, 26.49	1.00	2634
$SD_{BA.5\ NT}$	1.31	1.50	0.04, 5.31	1.00	2993
$SD_{anti\text{-}N}$	2.40	2.55	0.06, 9.08	1.00	2752
**Asymptomatic**					
Intercept	-3.48	0.09	-3.67, -3.30	1.00	2968
Log_{10} BA.5 NT	0.22	0.26	-0.27, 0.73	1.00	3051
Log_{10} anti-N	5.63	0.88	4.00, 7.52	1.00	2949
$SD_{BA.5\ NT}$	1.06	1.40	0.03, 4.88	1.00	2969
$SD_{anti\text{-}N}$	3.76	2.33	1.35, 9.97	1.00	2863

SD, standard derivation

Supplementary Table 9. Summary of the model estimates from re-infection risk estimation using anti-S and anti-N antibody titers in the hybrid immunity holders.

Parameter	Estimate	Standard Error	95% CI	Rhat	Effective sample size
Intercept	-4.19	0.06	-4.31, -4.07	1.00	3092
Log_{10} anti-S	-2.72	0.43	-3.57, -1.91	1.00	2788
Log_{10} anti-N	3.67	0.19	3.30, 4.03	1.00	2980
SD_{anti-S}	2.94	2.06	0.91, 8.22	1.00	3171
SD_{anti-N}	4.16	2.46	1.58, 10.80	1.00	2923

SD, standard derivation

Supplementary Table 10. Summary of the model estimates from re-infection risk estimation using BA.5 neutralizing and anti-N antibody titers in the hybrid immunity holders.

Parameter	Estimate	Standard Error	95% CI	Rhat	Effective sample size
Intercept	-5.12	0.32	-5.78, -4.54	1.00	2823
Log_{10} BA.5 NT	-7.57	1.92	-11.41, -3.97	1.00	3128
Log_{10} anti-N	3.12	0.82	1.57, 4.78	1.00	2752
$SD_{BA.5\ NT}$	5.24	2.90	1.97, 12.79	1.00	3033
$SD_{anti\text{-}N}$	1.86	1.80	0.08, 6.59	1.00	2720

SD, standard derivation

Supplementary Table 11. Summary of the model estimates from anti-N antibody response

Infection history	Parameter	Estimate	Standard Error	95% CI	Rhat	Effective sample size
Primary infection	h	3.16	0.00	3.02, 3.36	1.00	1416
Primary infection	α	0.59	0.00	0.41, 0.81	1.00	1425
Primary infection	β	0.04	0.00	0.02, 0.06	1.00	1409
Primary infection	λ	0.00	0.00	0.00, 0.00	1.00	1469
Primary infection	σ	0.56	0.00	0.54, 0.59	1.00	1581
Re-infection	h	3.09	0.00	2.76, 3.51	1.00	1532
Reinfection	α	0.47	0.01	0.02, 1.57	1.00	1733
Reinfection	β	0.51	0.01	0.05, 1.70	1.00	1557
Reinfection	λ	0.00	0.00	0.00, 0.00	1.00	1580
Reinfection	σ	0.52	0.00	0.39, 0.69	1.00	1612

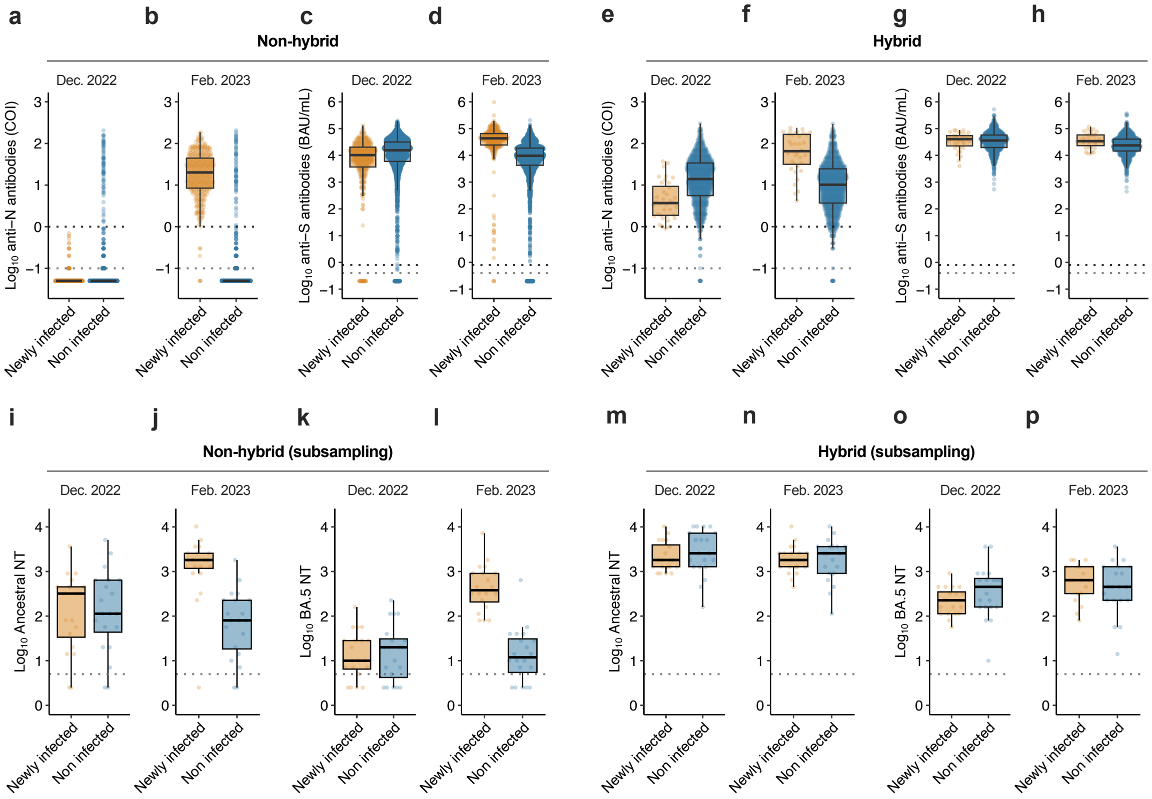

Supplementary Figure 1. Serum antibody levels in the presence or absence of new infections.

(a–d) The antibody levels for participants without hybrid immunity at baseline (Dec. 2022) and the end of observation (Feb. 2023). (a, b) Anti-N antibody levels and (c, d) anti-S antibody titers. n = 3,506. (e–h) The antibody levels for participants with hybrid immunity. (e, f) Anti-N antibody levels and (g, h) anti-S antibody titers. n = 990. (i–l) Neutralizing antibody titers in non-hybrid immunity holders at baseline (Dec. 2022) and the end of observation (Feb. 2023) against the SARS-CoV-2 ancestral strain (i, j) and Omicron BA.5 variant (k, l). n = 40. (m-p) Neutralizing antibody titers in hybrid immunity holders at baseline (Dec. 2022) and the end of observation (Feb. 2023) against the SARS-CoV-2 ancestral strain (m, n) and Omicron BA.5 variant (o, p). n = 40. Each data point (dots) and the box plots are shown. The dark gray dotted line indicates the cutoff value according to the manufacturer's manual, and the light gray dotted line represents the detection limit.

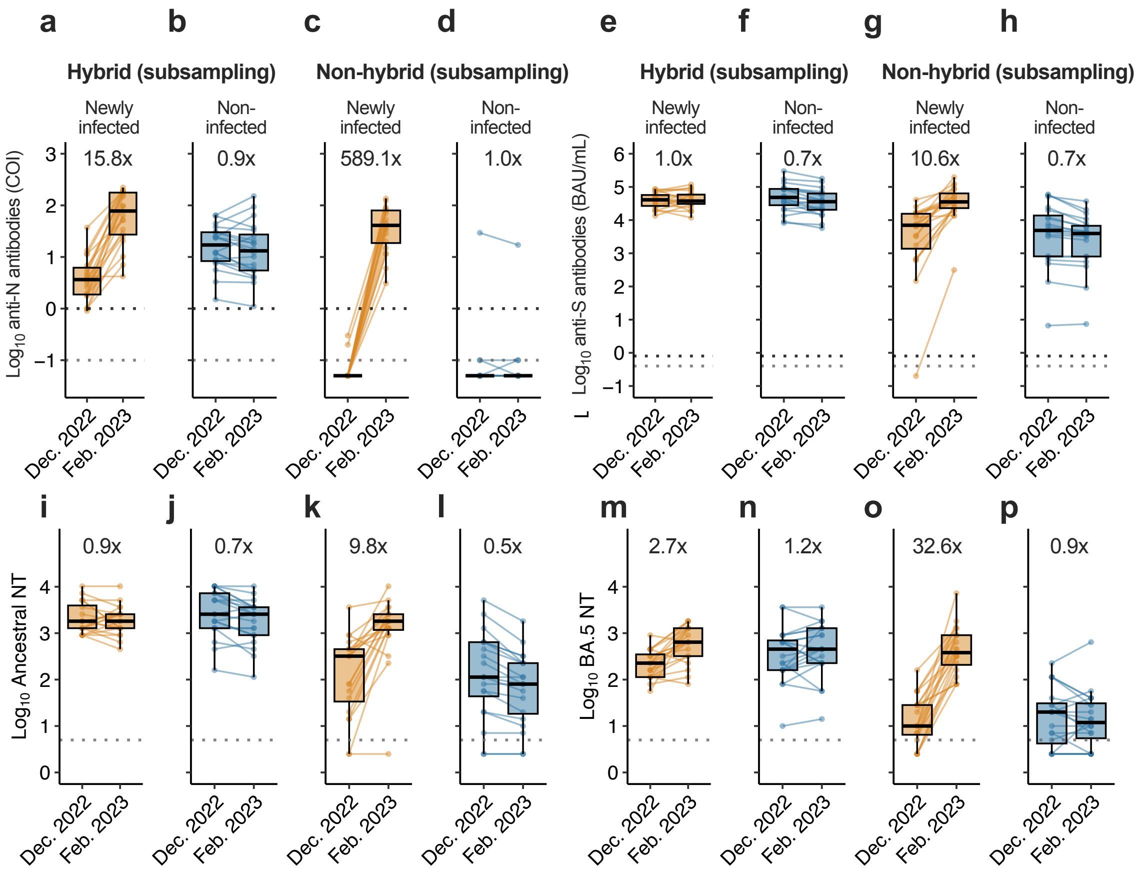

Supplementary Figure 2. Serum antibody responses before and after infection in subsampled specimens for neutralizing antibody response analysis.

(a–d) Anti-N antibody levels, (e–h) anti-S antibody titers, and neutralizing antibody titers against the ancestral strain (i–l) and the BA.5 variant (m–p) are shown for individuals with and without hybrid immunity, with and without new infections, at the time of the December 2022 survey and the February 2023 survey. Each data point (dots) and the box plots are shown. Data from the same participants are connected with lines. Fold increases are indicated above the columns. The dark gray dotted line indicates the cutoff value according to the manufacturer's manual, and the light gray dotted line represents the detection limit. n=80 participants.

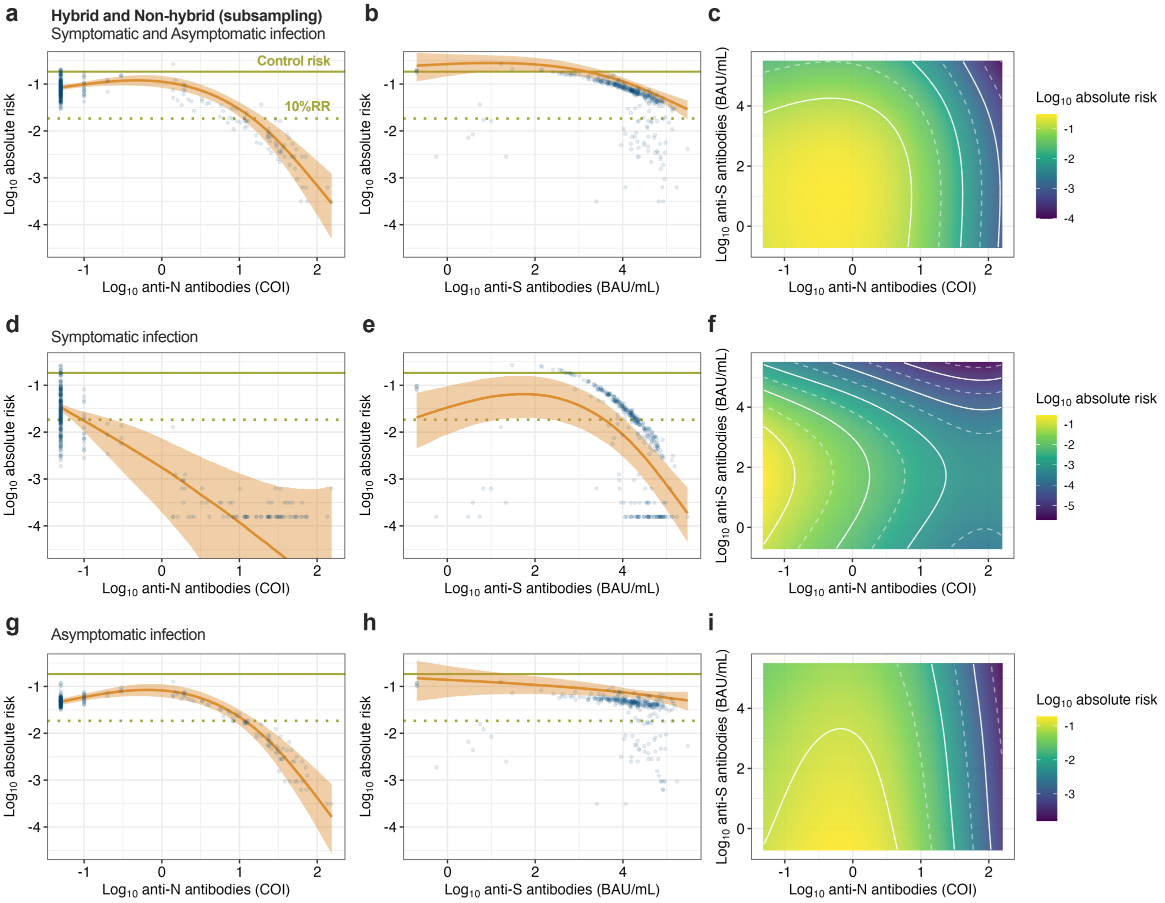

Supplementary Figure 3. Estimation of the effect of baseline anti-N and anti-S antibody levels on infection risk during the study period in the subsampling population. (a, b, d, e, g, h) The conditional effects of anti-N antibody levels and anti-S antibody titers during the observation period on the absolute risk of both symptomatic and asymptomatic infection (a, b), symptomatic infection (d, e), and asymptomatic infection (g, h), respectively. Each predicted data point (blue dots), along with the median absolute risk (line) and 95% CredIs (ribbon), are shown. The overall risk of a control group with no vaccination or prior infection history in this cohort (0.184) (green solid line) and the 90% reduction of relative risk (i.e., 10% relative risk [10%RR]) (green dotted line) are shown. (c, f, i) The combined effect of anti-N and anti-S antibody levels during the observation period on the absolute risk of both symptomatic and asymptomatic infection (c), symptomatic infection (f), and asymptomatic infection (i), respectively. The logarithmic absolute risk of infection is indicated by the color bar. The white dotted and solid lines show the $\log_{10}$ absolute risk decrease for every 0.5 and 1.0, respectively. Overall (a–c) and symptomatic infection (d–f); n=340. Asymptomatic infection (g–i); n=324.
