# Supplementary material for: Serum anti-nucleocapsid antibody correlates of protection from SARS-CoV-2 re-infection regardless of symptoms or immune history
Source: Commun Med (Lond). 2025 May 15;5:172. doi: 10.1038/s43856-025-00894-8 (PMC12081900; doi:10.1038/s43856-025-00894-8)
Supplement: Supplementary file 3 — Description of Additional Supplementary Files [file 43856_2025_894_MOESM3_ESM.docx]

Serum anti-nucleocapsid antibody correlates of protection from SARS-CoV-2
re-infection regardless of symptoms or immune history

Sho Miyamoto, Koki Numakura, Ryo Kinoshita, Takeshi Arashiro, Hiromizu Takahashi, Hiromi Hibino, Minako Hayakawa, Takayuki Kanno, Akiko Sataka, Rena Sakamoto, Akira Ainai, Satoru Arai, Motoi Suzuki, Daisuke Yoneoka, Takaji Wakita, Tadaki Suzuki

**Supplementary Data 1. Source Data for Figure 1 for primary infected individuals in anti-N antibody response model.**

**Supplementary Data 2. Source Data for Figure 1 for re-infected individuals in anti-N antibody response model.**

**Supplementary Data 3. Source Data for Figure 2-5 and Supplementary Figure 3.**

**Supplementary Data 4. Source Data for Figure 2f–v and Supplementary Figure 1–2.**

**Supplementary Data 5. Source Data for Figure 2o–v.**
