# Supplementary material for: Serum anti-nucleocapsid antibody correlates of protection from SARS-CoV-2 re-infection regardless of symptoms or immune history
Source: Commun Med (Lond). 2025 May 15;5:172. doi: 10.1038/s43856-025-00894-8 (PMC12081900; doi:10.1038/s43856-025-00894-8)
Supplement: Supplementary file 9 — Reporting Summary [file 43856_2025_894_MOESM9_ESM.pdf]

nature portfolio

Corresponding author(s): Tadaki Suzuki

Last updated by author(s): Apr 4, 2025

# Reporting Summary

Nature Portfolio wishes to improve the reproducibility of the work that we publish. This form provides structure for consistency and transparency in reporting. For further information on Nature Portfolio policies, see our Editorial Policies and the Editorial Policy Checklist.

## Statistics

For all statistical analyses, confirm that the following items are present in the figure legend, table legend, main text, or Methods section.

| n/a | Confirmed | |
|---|---|---|
| ☐ | ☒ | The exact sample size (*n*) for each experimental group/condition, given as a discrete number and unit of measurement |
| ☐ | ☒ | A statement on whether measurements were taken from distinct samples or whether the same sample was measured repeatedly |
| ☐ | ☒ | The statistical test(s) used AND whether they are one- or two-sided<br>*Only common tests should be described solely by name; describe more complex techniques in the Methods section.* |
| ☐ | ☒ | A description of all covariates tested |
| ☐ | ☒ | A description of any assumptions or corrections, such as tests of normality and adjustment for multiple comparisons |
| ☐ | ☒ | A full description of the statistical parameters including central tendency (e.g. means) or other basic estimates (e.g. regression coefficient) AND variation (e.g. standard deviation) or associated estimates of uncertainty (e.g. confidence intervals) |
| ☐ | ☒ | For null hypothesis testing, the test statistic (e.g. *F*, *t*, *r*) with confidence intervals, effect sizes, degrees of freedom and *P* value noted<br>*Give P values as exact values whenever suitable.* |
| ☐ | ☒ | For Bayesian analysis, information on the choice of priors and Markov chain Monte Carlo settings |
| ☒ | ☐ | For hierarchical and complex designs, identification of the appropriate level for tests and full reporting of outcomes |
| ☐ | ☒ | Estimates of effect sizes (e.g. Cohen's *d*, Pearson's *r*), indicating how they were calculated |

*Our web collection on statistics for biologists contains articles on many of the points above.*

## Software and code

Policy information about availability of computer code

| | |
|---|---|
| Data collection | cobas e411 (Roche), Microsoft® Excel for Mac 16.86 |
| Data analysis | Prism 9.5(Graphpad Software), R 4.3, rstan 2.26, brms 2.20 |

For manuscripts utilizing custom algorithms or software that are central to the research but not yet described in published literature, software must be made available to editors and reviewers. We strongly encourage code deposition in a community repository (e.g. GitHub). See the Nature Portfolio guidelines for submitting code & software for further information.

## Data

Policy information about availability of data

All manuscripts must include a data availability statement. This statement should provide the following information, where applicable:

- Accession codes, unique identifiers, or web links for publicly available datasets
- A description of any restrictions on data availability
- For clinical datasets or third party data, please ensure that the statement adheres to our policy

Source data for the main figures in the manuscript are provided in Supplementary Data files 1–5. The R codes for infection risk estimation and modeling the antibody response are available in a Zenodo Repository.

## Research involving human participants, their data, or biological material

Policy information about studies with human participants or human data. See also policy information about sex, gender (identity/presentation), and sexual orientation and race, ethnicity and racism.

| | |
|---|---|
| Reporting on sex and gender | Individuals who agreed to participate in the study answered a self-administered questionnaire that included information on biological sex. |
| Reporting on race, ethnicity, or other socially relevant groupings | All participants were Japanese. |
| Population characteristics | Population characteristics are shown in Supplementary Table 1. |
| Recruitment | Individuals who agreed to participate in the study answered a self-administered questionnaire, visited the designated site where they provided written consent, and had their blood drawn (See Methods). |
| Ethics oversight | All the samples, protocols, and procedures described herein were approved by the Medical Research Ethics Committee of the NIID and involved human participants, based on the principles of the Declaration of Helsinki (approval numbers 1457, 1472 and 1730). |

Note that full information on the approval of the study protocol must also be provided in the manuscript.

# Field-specific reporting

Please select the one below that is the best fit for your research. If you are not sure, read the appropriate sections before making your selection.

☒ Life sciences ☐ Behavioural & social sciences ☐ Ecological, evolutionary & environmental sciences

For a reference copy of the document with all sections, see nature.com/documents/nr-reporting-summary-flat.pdf

# Life sciences study design

All studies must disclose on these points even when the disclosure is negative.

| | |
|---|---|
| Sample size | We planned to enroll 15,000 individuals from five prefectures (3,000 individuals per prefecture). Assuming a response rate of 20%, we randomly sampled 75,000 individuals aged 20 years or older and invited them via mail. Only one participant from each household participated in the study. |
| Data exclusions | Of the 15,000 invitees for the national COVID-19 seroepidemiological survey conducted as a public health investigation, 8,157 participated in the December 2022 survey and 5,627 participated in the February to March 2023 survey (response rate: 37·5%; Fig. 1). We targeted the 5,627 individuals who participated in both surveys. Those who received any COVID-19 vaccine during the observation period were excluded (1,044 individuals) to eliminate the influence of the vaccination immune response during the study period. In addition, 87 individuals with a history of infection within 30 days before the initial test date were excluded (87 individuals) because the antibody response up to 30 days after infection is dynamic and difficult to accurately assess at this blood collection interval. Therefore, 4,496 individuals were enrolled for the ad hoc study. |
| Replication | All antibody titers were measured using commercial kits. There was no repeated measurement of antibody titers due to the large number of specimens. |
| Randomization | We planned to enroll 15,000 individuals from five prefectures (3,000 individuals per prefecture). Assuming a response rate of 20%, we randomly sampled 75,000 individuals aged 20 years or older and invited them via mail. |
| Blinding | In the exploratory ad hoc study, it was not possible to blind newly infected and non-newly infected participants. |

# Reporting for specific materials, systems and methods

We require information from authors about some types of materials, experimental systems and methods used in many studies. Here, indicate whether each material, system or method listed is relevant to your study. If you are not sure if a list item applies to your research, read the appropriate section before selecting a response.

## Materials & experimental systems

| n/a | Involved in the study |
|---|---|
| ☒ | ☐ Antibodies |
| ☐ | ☒ Eukaryotic cell lines |
| ☒ | ☐ Palaeontology and archaeology |
| ☒ | ☐ Animals and other organisms |
| ☒ | ☐ Clinical data |
| ☒ | ☐ Dual use research of concern |
| ☒ | ☐ Plants |

## Methods

| n/a | Involved in the study |
|---|---|
| ☒ | ☐ ChIP-seq |
| ☒ | ☐ Flow cytometry |
| ☒ | ☐ MRI-based neuroimaging |

# Eukaryotic cell lines

Policy information about cell lines and Sex and Gender in Research

| | |
|---|---|
| Cell line source(s) | VeroE6/TMPRSS2 cells (JCRB1819, Japanese Collection of Research Bioresources Cell Bank; Osaka, Japan) |
| Authentication | VeroE6/TMPRSS2 cells are registered in NIBSC and JCRB. |
| Mycoplasma contamination | JCRB confirms that elimitation of mycoplasma from VeroE6/TMPRSS2 was successfully done on 3-June-2020. |
| Commonly misidentified lines (See ICLAC register) | VeroE6/TMPRSS2 (Cellosaurus Accession No. CVCL_YQ49) are not listed in the misidentified lines on version 13. |

# Plants

| | |
|---|---|
| Seed stocks | *Report on the source of all seed stocks or other plant material used. If applicable, state the seed stock centre and catalogue number. If plant specimens were collected from the field, describe the collection location, date and sampling procedures.* |
| Novel plant genotypes | *Describe the methods by which all novel plant genotypes were produced. This includes those generated by transgenic approaches, gene editing, chemical/radiation-based mutagenesis and hybridization. For transgenic lines, describe the transformation method, the number of independent lines analyzed and the generation upon which experiments were performed. For gene-edited lines, describe the editor used, the endogenous sequence targeted for editing, the targeting guide RNA sequence (if applicable) and how the editor was applied.* |
| Authentication | *Describe any authentication procedures for each seed stock used or novel genotype generated. Describe any experiments used to assess the effect of a mutation and, where applicable, how potential secondary effects (e.g. second site T-DNA insertions, mosiacism, off-target gene editing) were examined.* |
